# Roles of microRNA-34a targeting SIRT1 in mesenchymal stem cells

**DOI:** 10.1186/s13287-015-0187-x

**Published:** 2015-10-07

**Authors:** Fengyun Zhang, Jinjin Cui, Xiaojing Liu, Bo Lv, Xinxin Liu, Zulong Xie, Bo Yu

**Affiliations:** Key Laboratories of Education Ministry for Myocardial Ischemia Mechanism, The Second Affiliated Hospital of Harbin Medical University, 148 Baojian Road, Harbin, 150086 P.R. China; Department of Cardiology, The Second Affiliated Hospital of Harbin Medical University, 148 Baojian Road, Harbin, 150086 P.R. China; Department of Cardiology, Mudanjiang Forestry Central Hospital, 50 XinhuaRoad, Mudanjiang, 157000 P.R. China

## Abstract

**Introduction:**

Mesenchymal stem cell (MSC)-based therapies have had positive outcomes both in animal models of cardiovascular diseases and in clinical patients. However, the number and function of MSCs decline during hypoxia and serum deprivation (H/SD), reducing their ability to contribute to endogenous injury repair. MicroRNA-34a (miR-34a) is originally identified as a TP53-targeted miRNA that modulates cell functions, including apoptosis, proliferation, and senescence via several signaling pathways, and hence is an appealing target for MSC-based therapy for myocardial infarction.

**Methods:**

Bone marrow-derived MSCs were isolated from 60–80 g male donor rats. Expression levels of miR-34a were determined by qRT-PCR. The roles of miR-34a in regulating cell vitality, apoptosis and senescence were investigated using the cell counting kit (CCK-8) assay, flow cytometric analysis of Annexin V-FITC/PI staining and senescence-associated β-galactosidase (SA-β-gal) staining, respectively. The expression of silent information regulator 1 (SIRT1) and forkhead box class O 3a (FOXO3a) and of apoptosis- and senescence-associated proteins in MSCs were analyzed by western blotting.

**Results:**

The results of the current study showed that miR-34a was significantly up-regulated under H/SD conditions in MSCs, while overexpression of miR-34a was significantly associated with increased apoptosis, impaired cell vitality and aggravated senescence. Moreover, we found that the mechanism underlying the proapoptotic function of miR-34a involves activation of the SIRT1/FOXO3a pathway, mitochondrial dysfunction and finally, activation of the intrinsic apoptosis pathway. Further study showed that miR-34a can also aggravate MSC senescence, an effect which was partly abolished by the reactive oxygen species (ROS) scavenger, N-acetylcysteine (NAC).

**Conclusions:**

Our study demonstrates for the first time that miR-34a plays pro-apoptotic and pro-senescence roles in MSCs by targeting SIRT1. Thus, inhibition of miR-34a might have important therapeutic implications in MSC-based therapy for myocardial infarction.

## Introduction

Ischemic heart disease (IHD) is the leading cause of death worldwide, and the resulting heart failure aggravates a country’s health burden, particularly in developed countries [1]. Existing therapies are typically only able to slow, rather than reverse or prevent, the progression of heart failure. Furthermore, side effects remain the key issue among these effective therapeutics [2]. In the last few years, bone marrow-derived mesenchymal stem cells (MSCs) have been found to function as one of the most suitable candidate seed cells for repairing and regenerating cardiomyocytes as well as restoring heart function, and have been widely studied [3, 4]. Transplantation of MSCs leads to improved neovascularization of ischemic myocardium and inhibition of myocardial fibrosis, in addition to an increase in the secretion of prosurvival growth factors, including vascular endothelial growth factor, insulin-like growth factor, and hepatocyte growth factor [4, 5]. Despite these advantages, the poor survival rate of MSCs within the first few days after engrafting in infarcted hearts leads to only marginal functional improvement [6, 7]. The harsh microenvironment of the infarcted myocardium produces high levels of oxidative stress, which makes a great contribution to cellular senescence and causes a sharp decline in the proliferative capacity and regenerative potential of MSCs [8]. There is thus an urgent need to identify a strategy to protect the cells against the hostile microenvironment created by ischemia, hypoxia, the inflammatory response, and pro-apoptotic and pro-senescence factors in order to improve the efficacy of MSC transplantation therapy.

MicroRNAs (miRNAs) are endogenous ~22-nucleotide RNAs that have emerged as negative regulators of gene expression, acting by targeting mRNAs for cleavage or translational repression, which occurs primarily through base pairing to the 3′ untranslated regions (UTRs) of target mRNAs [9, 10]. With rapid advances in understanding of the regulation and roles of these small, noncoding RNAs in cardiac pathology, the therapeutic potential of regulation of miRNAs in cardiac disease settings is considered high [9, 11]. Among the known miRNAs, expression of miR-34a was found to be elevated in mouse hearts after myocardial infarction (MI) [12] and in cardiac tissue from patients with heart disease [13], while inhibition of the expression of miR-34a alleviated apoptosis and senescence in myocardial cells [14, 15] and other cell lines [16–18]. However, the precise role of miR-34a in MSCs has not been unraveled to date.

Silent information regulator 1 (SIRT1), one of the potential targets of miR-34a [19], is an NAD-dependent deacetylase that regulates apoptosis in response to oxidative and genotoxic stress and plays a critical role in regulating cell cycle, senescence, and metabolism [19–21]. Initially identified as a longevity gene, SIRT1 has recently been implicated as a novel modulator of myocyte homeostasis, playing a key role in cardiomyopathy through the deacetylation of forkhead box O transcription factor 3a (FOXO3a) [20], which was also acknowledged as the transcription factor most closely related to the anti-oxidative protective effects associated with longevity [22, 23]. Further study showed in endothelial progenitor cells (EPCs) that SIRT1 has a pivotally protective role in the regulation of EPC apoptosis induced by H_2_O_2_, and that SIRT1 exerted this protective effect by inhibiting FOXO3a via FOXO3a ubiquitination and subsequent degradation [24]. However, it is entirely unknown whether SIRT1 affects biological activities in MSCs; and if so, what role FOXO3a plays in this process.

In the current study, we tested the hypothesis that overexpression of miR-34a increases cellular susceptibility to hypoxia and serum deprivation (H/SD)-induced apoptosis and aggravates cell senescence, and investigated the underlying mechanisms. The results showed that miR-34a played a crucial role in a plethora of biological processes via regulation of SIRT1/FOXO3a and the reactive oxygen species (ROS) pathway in MSCs. Inhibition of miR-34a might therefore be a promising therapeutic strategy for enhancing the biological functions of MSCs, thus demonstrating great therapeutic potential in clinical transplantation.

## Materials and methods

### Ethics statement

Male Sprague–Dawley rats weighing 60–80 g were obtained from the Laboratory Animal Science Department of the Second Affiliated Hospital of Harbin Medical University, Heilongjiang, P.R. China. All experimental animal procedures were approved by the Local Ethical Committee on Animal Care and Use of Harbin Medical University.

### MSC culture

MSCs were cultured using the whole bone marrow adherent method, as described previously [25]. Briefly, total bone marrow was harvested from the femora of rats and was plated into 25 cm^2^ culture flasks at a concentration of 10^6^ cells/ml in Iscove’s modified Dulbecco’s medium (IMDM; HyClone-Thermo Fisher Scientific, Waltham, MA, USA) supplemented with 10 % fetal bovine serum (FBS; Gibco, Grand Island, NY, USA) and 1 % penicillin/streptomycin (Beyotime Institute of Biotechnology, Nantong, China), at 37 °C with 5 % CO_2_. After 3 days of incubation, the medium was changed and then replaced every 3 days thereafter. Approximately 7–9 days after seeding, the cells became 70–80 % confluent. The adherent cells were released from the dishes using 0.25 % trypsin (Beyotime Institute of Biotechnology, Beijing, China) and expanded at a 1:2 or 1:3 dilution. MSCs at passage 3–5 were used in all experiments. MSCs were characterized by flow cytometric analysis for the expression of the typical markers CD90, CD29, and CD44 (all from BD Biosciences, Franklin Lakes, NJ, USA), and the absence of the hematopoietic markers CD45 (eBioscience, San Diego, CA, USA) and CD34 (Santa Cruz Biotechnology, Inc., Dallas, TX, USA), as reported previously [25].

### Cell viability assay

The viability of MSCs was determined using the cell counting kit-8 (CCK-8) assay (Beyotime Institute of Biotechnology, Beijing, China) in accordance with the manufacturer’s protocols. Cells were seeded into a 96-well plate (3000 cells per well), and their growth was measured following addition of 10 μl CCK-8 into the culture medium for 2 hours. The absorbance of each well was quantified at 450 nm (Tecan Infinite M200 microplate reader; LabX, Austria). All data were calculated from triplicate samples.

### MSC H/SD treatment

Apoptosis was induced by H/SD in vitro, which was designed to mimic the in vivo conditions of ischemia in the myocardium and was carried out as reported previously [26]. Briefly, MSCs were washed and cultured with serum-free IMDM and incubated in a 5 % CO_2_/95 % N_2_ incubator (controlled atmosphere chamber; PLAS-Labs, Lansing, MI, USA) for 6 hours. MSCs incubated in a 5 % CO_2_/95 % O_2_ incubator were used as the normoxic control and cultured in complete medium.

### Measurement of apoptosis

Apoptosis was determined by staining cells with Annexin V–fluorescein isothiocyanate (FITC) and counterstaining with propidium iodide (PI) using the Annexin V–FITC/PI apoptosis detection kit (BD PharMingen, San Diego, CA, USA). Briefly, 0.5 × 10^6^ cells were washed twice with phosphate-buffered saline (PBS) and stained with 5 μl Annexin V–FITC and 5 μl PI in 1× binding buffer (BD PharMingen) for 15 minutes at room temperature in the dark. Analyses were performed using bivariate flow cytometry in a BD FACSCanto II equipped with BD FACSDiva software (Becton-Dickinson, San Jose, CA, USA).

### Target gene prediction

To identify the potential targets of miR-34a that mediated its pro-apoptotic role in MSCs, bioinformatics algorithms including miRBase (University of Manchester, Manchester, UK), TargetScan (David Bartel Lab, Whitehead Institute for Biomedical Research, MA, USA), PicTar (Rajewsky lab, NY, USA and Max Delbruck Centrum, Berlin, DE), and miRanda (Computational Biology Center at MSKCC, NY, USA) were applied.

### Cell transfection

Before transfection, MSCs were replanted into six-well plates at a density of 2 × 10^5^ cells per well and incubated overnight. For overexpression or inhibition of miR-34a, cells were transfected with different concentrations of miR-34a mimic or miR-34a inhibitor (both from Invitrogen, Carlsbad, CA, USA). For small interfering RNA (siRNA)-mediated gene knockdown, 100 nM SIRT1 siRNA (GenePharma Co., Ltd, Shanghai, China) was transfected into cells. As controls, cells were transfected with negative control (NC) mimic, NC inhibitor of miR-34a (both from Invitrogen,Carlsbad, CA, USA), or scrambled siRNA (siRNA-NT) of SIRT1 (GenePharma Co., Ltd, Shanghai, China). All miRNAs and siRNA were transfected into MSCs using a commercial transfection reagent (X-treme siRNA Transfection Reagent; Roche Applied Science, Penzberg, Germany) according to the manufacturer’s protocol. Forty-eight or 72 hours after transfection, cells were harvested for further analysis.

### RNA extraction and quantitative RT-PCR

For analysis of miR-34a expression, total RNA was extracted from the MSCs using TRizol reagent (Invitrogen, Shanghai, China) and reverse-transcribed into cDNA according to the manufacturer’s instructions. Quantitative RT-PCR (qRT-PCR) was performed to analyze the level of miR-34a with the miRcute miRNA First-Strand cDNA Synthesis Kit and the miRcute miRNA qPCR Detection Kit (SYBR Green; Tiangen, Beijing, China). All primers for miR-34a and U6 for the TaqMan miRNA assays were purchased from Gene Pharma. Relative gene expression levels were calculated by comparing the △Ct values between control and experimental conditions for each PCR target using the following equation:

$$ \mathrm{Relative}\kern0.5em \mathrm{gene}\kern0.5em \mathrm{expression}={2}^{\hbox{-} \left(\Delta \mathrm{C}\mathrm{t}\kern0.5em \mathrm{sample}\hbox{-} \Delta \mathrm{C}\mathrm{T}\kern0.5em \mathrm{control}\right)} $$

For several other genes, total cellular RNA was isolated and reverse-transcribed using the transcriptor First-Stand cDNA Synthesis Kit, according to the manufacturer’s instructions. qRT-PCR was carried out using the fast-start universal SYBR master and fluorescence quantitative PCR system [27]. The relative expression level of mRNAs was normalized to that of the internal control glyceraldehyde 3-phosphate dehydrogenase (GAPDH) using the 2^–ΔΔCt^ cycle threshold method. Table 1 presents all related gene sequences.

**Table 1:** Primers for quantitative RT-PCR and oligonucleotides

Name		Sequence
Quantitative RT-PCR		
miR-34a	Forward	5′-AAGGCCACGGATAGGTCCATA-3′
	Reverse	5′-CGCTTTGGTGGTTCTGAAAGG-3′
SIRT1	Forward	5′-AAGGCCACGGATAGGTCCATA-3′
	Reverse	5′-CGCTTTGGTGGTTCTGAAAGG-3′
FOXO3a	Forward	5′-TGCCGATGGGTTGGATTT-3′
	Reverse	5′-CCAGTGAAGTTCCCCACGTT-3′
U6	Forward	5′-AAGGCCACGGATAGGTCCATA-3′
	Reverse	5′-CGCTTTGGTGGTTCTGAAAGG-3′
β-actin	Forward	5′-CCCAGCACAATGAAGATCAAGATCAT-3′
	Reverse	5′-ATCTGCTGGAAGGTGTACAGCGA-3′
Oligonucleotide		
miR-34a mimic		UGGCAGUGUCUUAGCUGGUUGUU CAACCAGCUAAGACACUGCCAUU
NC mimic		UUCUCCGAACGUGUCACGUTT ACGUGACACGUUCGGAGAATT
miR-34a inhibitor		UGGCAGUGUCUUAGCUGGUUGUU
NC inhibitor		CAGUACUUUUGUGUAGUACAA
siRNA-SIRT1		GCACCGAUCCUCGAACAAUTT AUUGUUCGAGGAUCGGUGCTT
siRNA-NT		UUCUCCGAACGUGUCACGUTT ACGUGACACGUUCGGAGAATT

*FOXO3a* forkhead box O transcription factor 3a, *miRNA* microRNA, *NC* negative control, *siRNA* small interfering RNA, *siRNA-NT* scrambled siRNA, *SIRT1* silent information regulator 1

### Measurement of mitochondrial membrane potential

Mitochondrial membrane potential (∆Ψm) was measured using the JC-1 mitochondrial membrane potential assay kit (Beyotime Institute of Biotechnology, Beijing, China). JC-1 was widely used to assess changes in ∆Ψm and mitochondrial permeability transition. After designated treatment, cells were incubated with JC-1 working dye for 20 minutes, then washed twice with cold JC-1 staining buffer and visualized under a fluorescence microscope (DMI4000B; Leica, Wetzlar, Germany).

### ROS staining

Cells were left untreated or pretreated with NAC, miR-34a mimic, siRNA-SIRT1, and miR-34a inhibitor separately or in combination and then stimulated with the diluted fluoroprobe 2′,7′-dichlorodihydrofluorescein diacetate (DCFH-DA; Beyotime Institute of Biotechnology, Beijing, China) for 20 minutes at 37 °C with slight shaking every 5 minutes. After washing with serum-free culture medium, the cells were collected and examined by flow cytometry.

### Senescence-associated β-galactosidase staining

MSC senescence was determined by in situ staining for senescence-associated β-galactosidase (SA-β-gal) using a senescence cell histochemical staining kit (Beyotime Institute of Biotechnology, Beijing, China). Briefly, MSCs after treatment were first fixed for 30 minutes at room temperature in fixation buffer. After washing with PBS, cells were incubated with β-galactosidase staining solution for 16 hours at 37 °C without CO_2_. The reaction was stopped by the addition of PBS. Statistical analysis was performed by counting 600 cells for each sample.

### Protein extraction and western blot analysis

After designated treatment, cells were washed twice with ice-cold PBS, and the total protein concentration was analyzed using the bicinchoninic acid assay (BCA; Beyotime Institute of Biotechnology, Beijing, China) according to the manufacturer’s instructions. Total cell extracts (50 μg total protein) were resolved by sodium dodecyl sulfate (SDS)–10 % polyacrylamide gel electrophoresis and transferred onto polyvinylidene difluoride (PVDF) membranes. Nonspecific binding was inhibited by incubating the membranes with 8 % skimmed milk in Tris-buffered saline (TBS) with 0.5 % Tween-20. Subsequently, membranes were incubated with antibodies against SIRT1, FOXO3a, cleaved-caspase 3 (Cl.CASP3), cleaved-polyADP-ribose polymerase 1 (Cl.PARP1), cytochrome *c*, P53 (all from Cell Signaling Technology, Danvers, MA, USA), p16, γ-H2A.X (both from Abcam, Cambridge, MA, USA), p21 (Santa Cruz, CA, USA), and β-actin (Zhongshan Golden Bridge Biotechnology, Beijing, China) overnight at 4 °C at an appropriate dilution (1:1000). The membranes were washed with TBS with Tween-20 (TBS-T) and then incubated with peroxidase-conjugated Affinipure goat anti-rabbit IgG (H + L) and anti-mouse IgG (H + L)-labeled secondary antibodies (Zhongshan Golden Bridge Biotechnology, Beijing, China) diluted at 1:5000 for 1 hour at 37 °C. Specific complexes were visualized on an X-ray film using Electro-Chemi-Luminescence (ECL) detection with BeyoECL Plus (Beyotime Institute of Biotechnology, Beijing, China) following the manufacturer’s protocol. All data were obtained in triplicate, independent experiments.

### Statistical analysis

All data were analyzed using SPSS 19.0 (SPSS Inc., Chicago, IL, USA) and were expressed as mean ± standard deviation (SD). Comparisons between two groups were performed using Student’s *t* test, while the significance of differences between three or more experimental groups was determined by one-way analysis of variance. *P* <0.05 was considered statistically significant.

## Results

### miR-34a expression increases under H/SD, and correlates with decreased cell survival and increased apoptosis

**Fig. 1:**
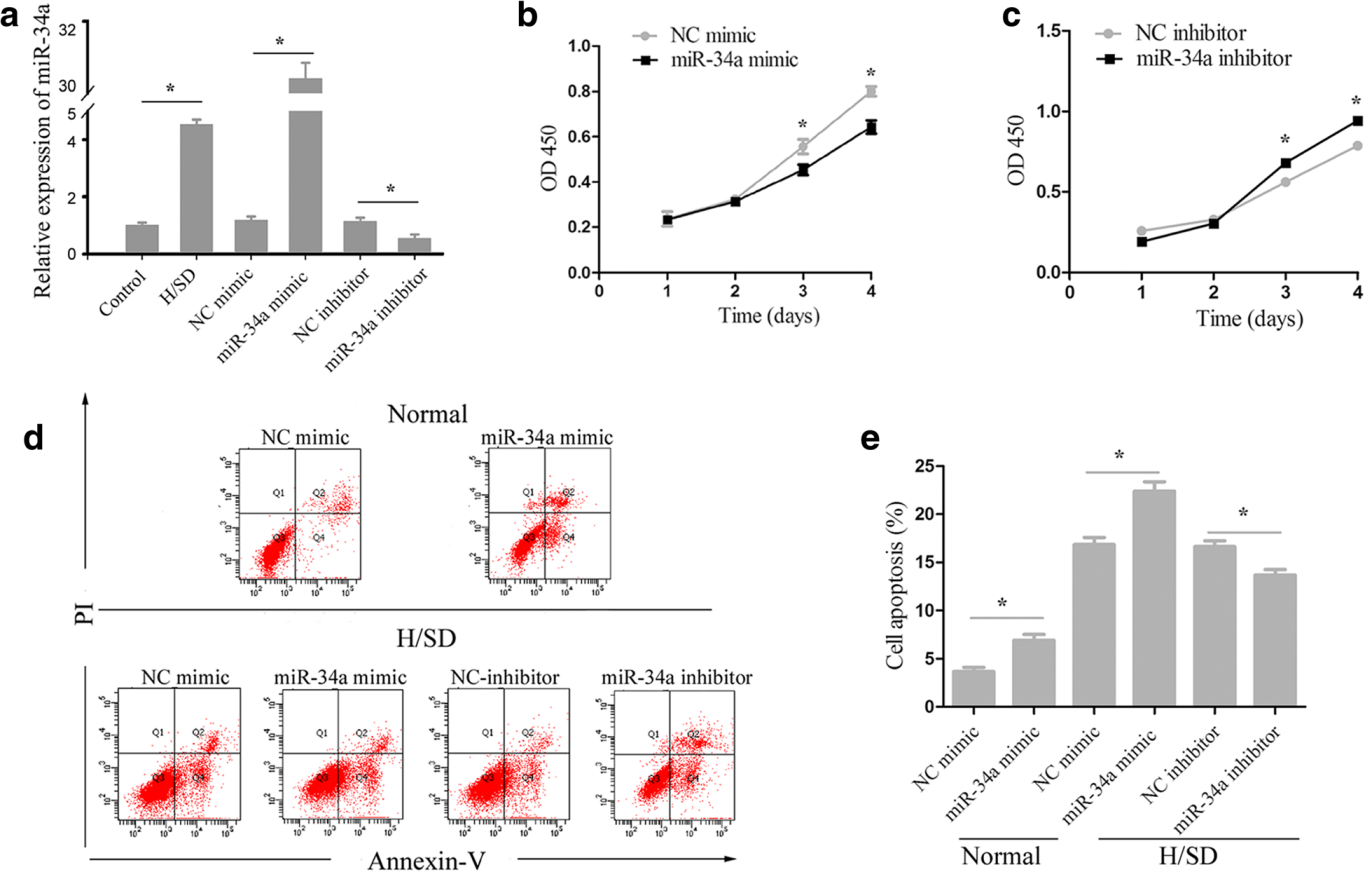
miR-34a expression increases under H/SD, and correlates with decreased cell survival and increased apoptosis. **a** Rat MSCs were cultured in normal condition or exposed to H/SD for 6 hours and were transiently transfected with miR-34a mimic, NC mimic, miR-34a inhibitor, or NC inhibitor for 48 hours, respectively. The expression of miR-34a was determined by qRT-PCR. *P <0.05. **b**, **c** Effects of miR-34a on the vitality of MSCs were examined by CCK-8 assay. *P <0.05 vs. NC mimic (**b**), *P <0.05 vs. NC inhibitor (**c**). **d**, **e** Flow cytometric analysis of apoptotic cells in normal and H/SD conditions, in cultures of miR-34a mimic, NC mimic, miR-34a inhibitor, or NC inhibitor treated (MSCs were transfected for 48 hours and exposure to H/SD and maintained as such for 6 hours). Each column represents mean ± SD from three independent experiments. *P <0.05. *H/SD* hypoxia and serum deprivation, *miRNA* microRNA, *NC* negative control, *PI* propidium iodide

qRT-PCR results showed that miR-34a was expressed in normal MSCs and that expression increased significantly when exposed to atmospheric conditions representing H/SD for 6 hours (Fig. 1a). We then used CCK-8 to evaluate the role of miR-34a in MSC survival, and found that overexpression of miR-34a reduced cell survival, while inhibition of miR-34a expression showed the opposite effect (Fig. 1b, c). Apoptosis has been identified as a major mechanism that reduces the survival rate after transplantation of MSCs into the harsh microenvironment of infarcted myocardium [26, 28]. To further determine the role of miR-34a in MSCs under conditions of H/SD, Annexin V–FITC/PI staining was performed. The results showed that miR-34a mimic-treated MSCs were significantly more apoptotic than the NC mimic group both in normal and H/SD conditions (Fig. 1d, e). However, when miR-34a was inhibited, MSCs showed better resistance to H/SD than the NC inhibitor group (Fig. 1d, e). This supports our hypothesis that the decreased cell survival and increased apoptosis in MSCs are associated with overexpression of miR-34a.

### SIRT1 is a direct target of posttranscriptional repression by miR-34a

**Fig. 2:**
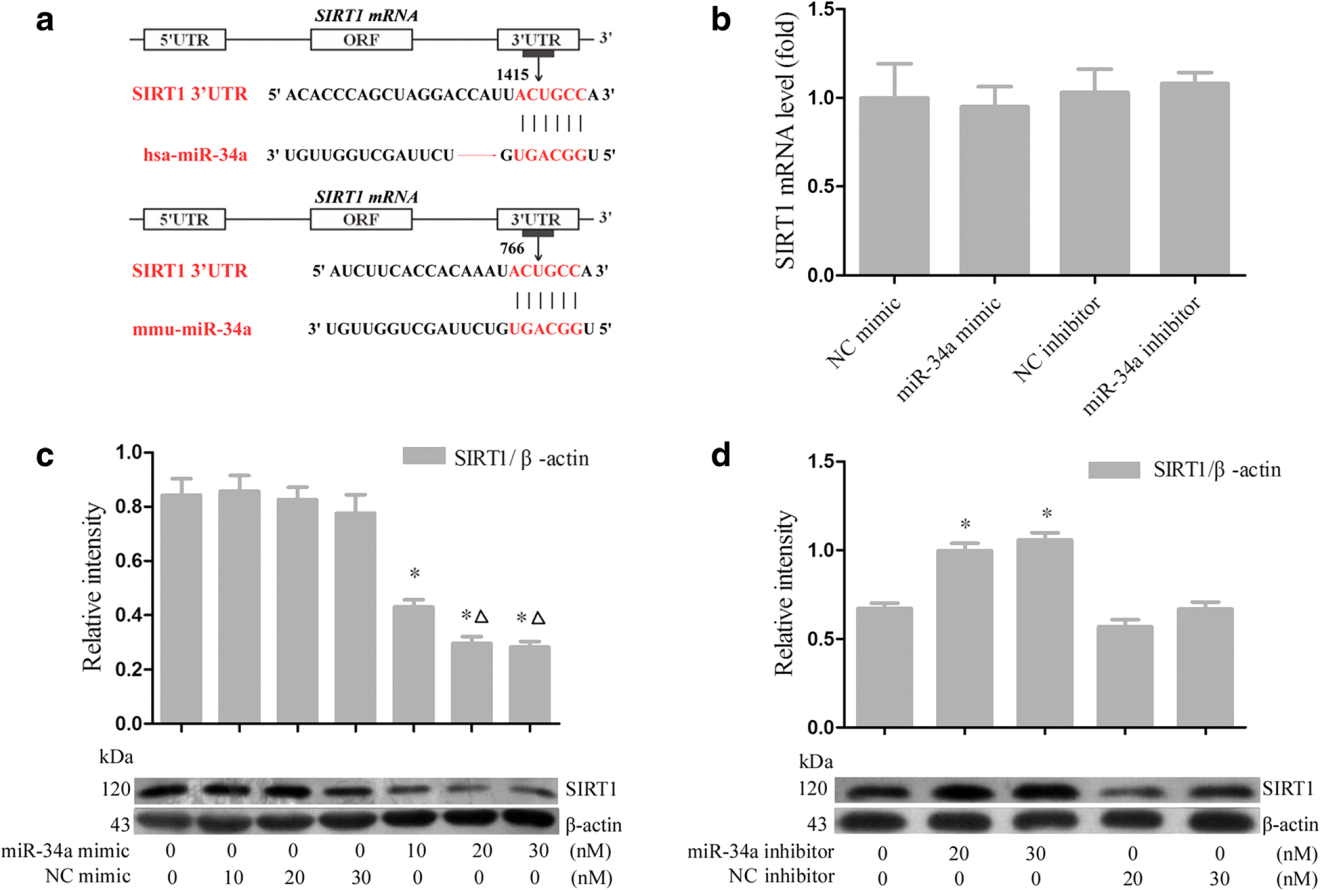
SIRT1 is a direct target of posttranscriptional repression by miR-34a. **a** The predicted targeting sites with miR-34a of SIRT1 3′-UTR (*Hsa*, human; *Mmu*, mouse) are highlighted in red. **b** qRT-PCR analysis was applied to detect mRNA expression of SIRT1 in MSCs after transfection with miR-34a mimic, NC mimic, miR-34a inhibitor, or NC inhibitor for 48 hours, respectively. **c**, **d** Western blot analysis showed dose-dependent regulation of SIRT1 by miR-34a after transfection with miR-34a mimic, NC mimic, miR-34a inhibitor, or NC inhibitor for 72 hours, respectively. Each column represents mean ± SD from three independent experiments. *P <0.05 vs. control, △*P* <0.05 vs. transfection with 10 nM miR-34a mimic. *miRNA* microRNA, *NC* negative control, *ORF* open reading frame, *SIRT1* silent information regulator 1, *UTR* untranslated region

Bioinformatics results suggested that SIRT1, identified as an apoptosis-associated gene, was the potential target of miR-34a. In addition, miRBase showed that the binding sites of miR-34a are evolutionarily conserved in both human and mouse (Fig. 2a). To test the hypothesis that miR-34a regulates SIRT1 expression in MSCs from rats, we transfected MSCs with a miR-34a mimic or miR-34a inhibitor. As a control, some cells were transfected with NC mimic or NC inhibitor. Western blot analysis demonstrated a dose-dependent decrease in SIRT1 protein expression in miR-34a mimic-transfected cells compared with the NC mimic group (Fig. 2c). Notably, the inhibition of miR-34a in MSCs was concurrent with the increased expression of SIRT1 (Fig. 2d). However, qRT-PCR showed little difference in the expression levels of SIRT1 mRNA among the treatment groups. These data suggest that SIRT1 is likely to be targeted by miR-34a posttranscriptionally.

### miR-34a induces apoptosis by modifying SIRT1 and FOXO3a expression

**Fig. 3:**
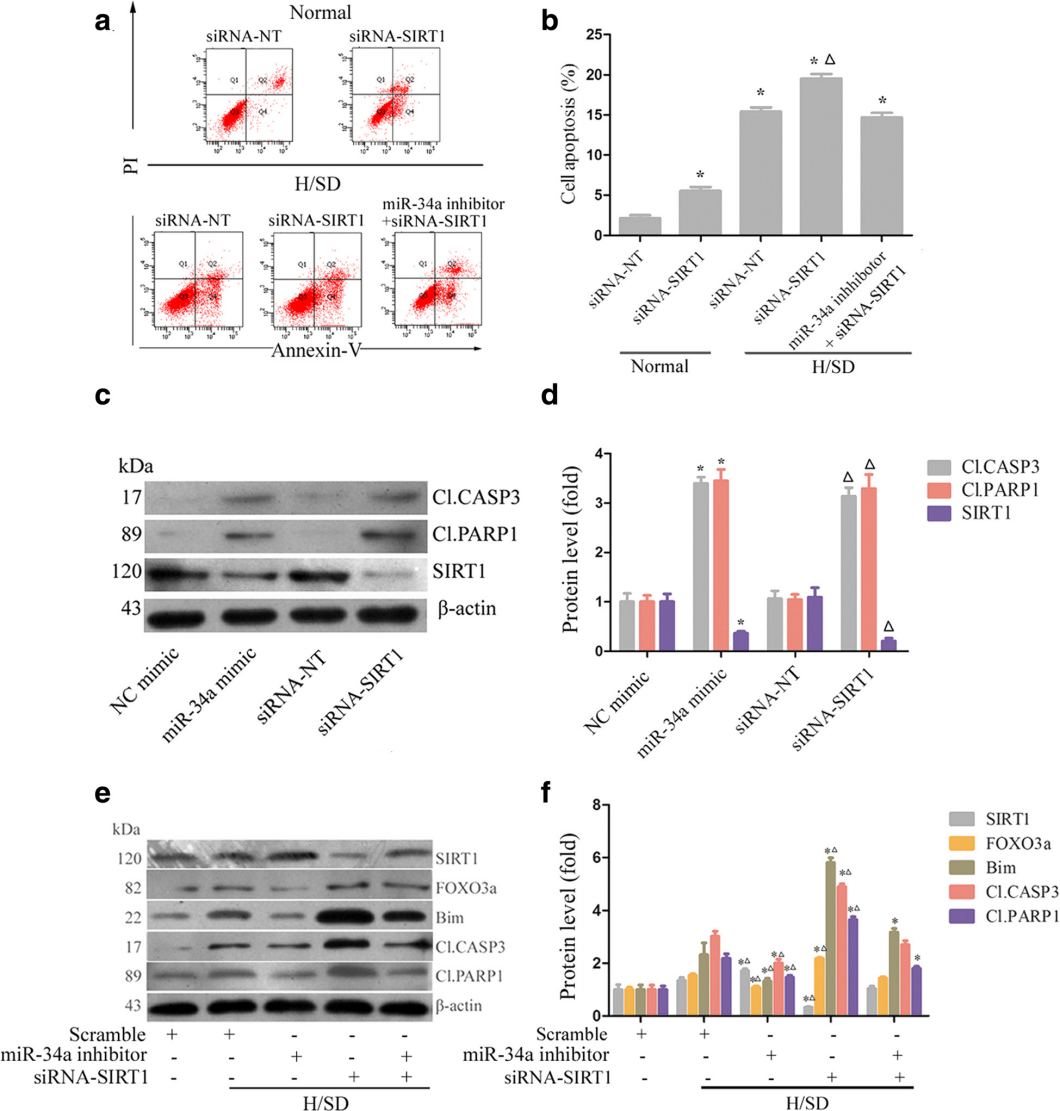
miR-34a induces apoptosis by modifying SIRT1 and FOXO3a expression. **a**, **b** Apoptosis was analyzed by measuring Annexin V^+^/PI^–^ cells using flow cytometry in cultures of siRNA-SIRT1, siRNA-NT, or siRNA-SIRT1 cotransfected with miR-34a inhibitor-treated MSCs, under normal and H/SD conditions (MSCs were transfected for 72 hours and exposure to H/SD and maintained as such for 6 hours). *P <0.05 vs. normal siRNA-NT, △*P* < 0.05 vs. H/SD siRNA-NT. **c**, **d** MSCs were transfected with miR-34a mimic, NC mimic, siRNA-SIRT1, or siRNA-NT for 72 hours, respectively, and then CASP3 and PARP1 activity was measured using western blot. *P <0.05 vs. NC mimic, △*P* <0.05 vs. siRNA-NT. **e**, **f** Western blot analysis of SIRT1, FOXO3a, Bim, CASP3, and PARP1 protein expression in cultures of siRNA-NT, siRNA-SIRT1, miR-34a inhibitor, or siRNA-SIRT1 cotransfected with miR-34a inhibitor-treated MSCs, under normal and H/SD conditions (MSCs were transfected for 72 hours and exposure to H/SD and maintained as such for 6 hours). β-actin was used as the internal control. Each column represents mean ± SD from three independent experiments. *P <0.05 vs. normal scramble, △*P* <0.05 vs. H/SD scramble. *CASP3* caspase 3, *FOXO3a* forkhead box O transcription factor 3a, *H/SD* hypoxia and serum deprivation, *miRNA* microRNA, *NC* negative control, *PARP1* polyADP-ribose polymerase 1, *PI* propidium iodide, *SIRT1* silent information regulator 1, *siRNA* small interfering RNA, *siRNA-NT* scrambled siRNA

After identifying SIRT1 as a direct target of miR-34a, we investigated whether knockdown of SIRT1 by siRNA (siRNA-SIRT1) induces apoptosis in MSCs. Similar to miR-34a mimic treatment, suppression of SIRT1 expression promoted apoptosis, revealed by flow cytometric analysis of the percentage of cells that were Annexin V^+^/PI^–^ (Fig. 3a, b). CASP3 is a well-studied mediator of apoptosis, because it is either partially or totally responsible for the cleavage of many key proteins, such as PARP1 [18]. In this study, increased activities of the cleaved CASP3 and cleaved PARP1 were observed when SIRT1 was knocked down or miR-34a was overexpressed (Fig. 3b, d). These findings suggest that knockdown of SIRT1 or treatment with miR-34a mimic acts similarly in the regulation of apoptosis.

To further elaborate the relationship between miR-34a and SIRT1, MSCs were transfected with miR-34a inhibitor or siRNA-SIRT1, or a combination, before exposure to H/SD. The results showed miR-34a inhibitor reduced CASP3 activity and expression of cleaved PARP1 (Fig. 3e, f), while siRNA-SIRT1 partly abolished the effects of the miR-34a inhibitor, verified both by flow cytometric analysis of the percentage of Annexin V^+^/PI^–^ cells (Fig. 3a, b) and by western blot analysis of cleaved CASP3 and cleaved PARP1 (Fig. 3e, f).

SIRT1 plays important roles in many pathophysiological processes by deacetylating various substrates, including FOXO3, which has been reported to promote apoptosis by regulating its downstream target, the well-known pro-apoptotic protein Bim [24]. Our work revealed that downregulation of miR-34a decreased total FOXO3a and Bim protein expression, whereas SIRT1 knockdown increased the expression of these two proteins (Fig. 3e, f). However, neither miR-34a inhibitor nor siRNA-SIRT1 altered the mRNA level of FOXO3a (data not shown), indicating that miR-34a and SIRT1 could regulate FOXO3a posttranscriptional activity under H/SD conditions. Our study revealed that the activation of SIRT1/FOXO3a and the intrinsic apoptosis pathway of CASP3–PARP1 might be involved in the pro-apoptotic function of miR-34a.

### miR-34a exerts pro-apoptotic effects via activation of the mitochondrial apoptosis pathway

**Fig. 4:**
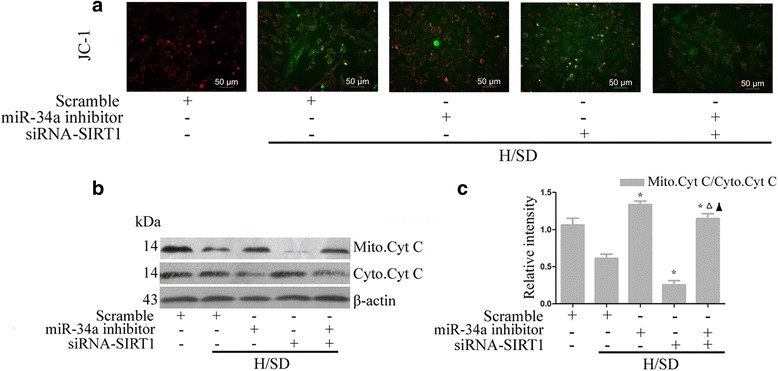
miR-34a exerts pro-apoptotic effects via activation of the mitochondrial apoptosis pathway. MSCs were transfected with siRNA-NT, siRNA-SIRT1, miR-34a inhibitor, or siRNA-SIRT1 cotransfected with miR-34a inhibitor under normal and H/SD conditions (MSCs were transfected for 72 hours and exposure to H/SD and maintained as such for 6 hours). Then ∆Ψm (**a**) was analyzed by measuring JC-1 fluorescence, and cytosolic and mitochondrial cytochrome *c* expression (**b**, **c**) were measured with western blot. Each column represents mean ± SD from three independent experiments. *P <0.05 vs. H/SD scramble, △*P* <0.05 vs. miR-34a inhibitor, ▲*P* <0.05 vs. siRNA-SIRT1. *H/SD* hypoxia and serum deprivation, *miRNA* microRNA, *SIRT1* silent information regulator 1, *siRNA* small interfering RNA

∆Ψm is the main parameter of mitochondrial function used as an indicator of cell health [29]. Therefore, to understand the intrinsic apoptotic pathway that is activated by miR-34a, we performed JC-1 staining. In contrast to the scramble cells, cells treated with the miR-34a inhibitor displayed significant changes in ΔΨm (Fig. 4a). To further ascertain the effect of miR-34a, cellular fractionation was performed and cell lysates from cytosolic and mitochondrial fractions were subjected to western blotting to detect the expression of cytochrome *c*, which was released from mitochondria and functioned as a key mediator of apoptosis [30]. Western blot analysis revealed an inhibition of cytochrome *c* release in the miR-34a inhibitor group, while siRNA-SIRT1 reversed its effect (Fig. 4b, c). Taken together, these data support the hypothesis that miR-34a may be involved in the apoptotic process of MSCs induced by H/SD through activation of the mitochondrial apoptosis pathway by targeting SIRT1.

### Overexpression of miR-34a induces senescence in MSCs

**Fig. 5:**
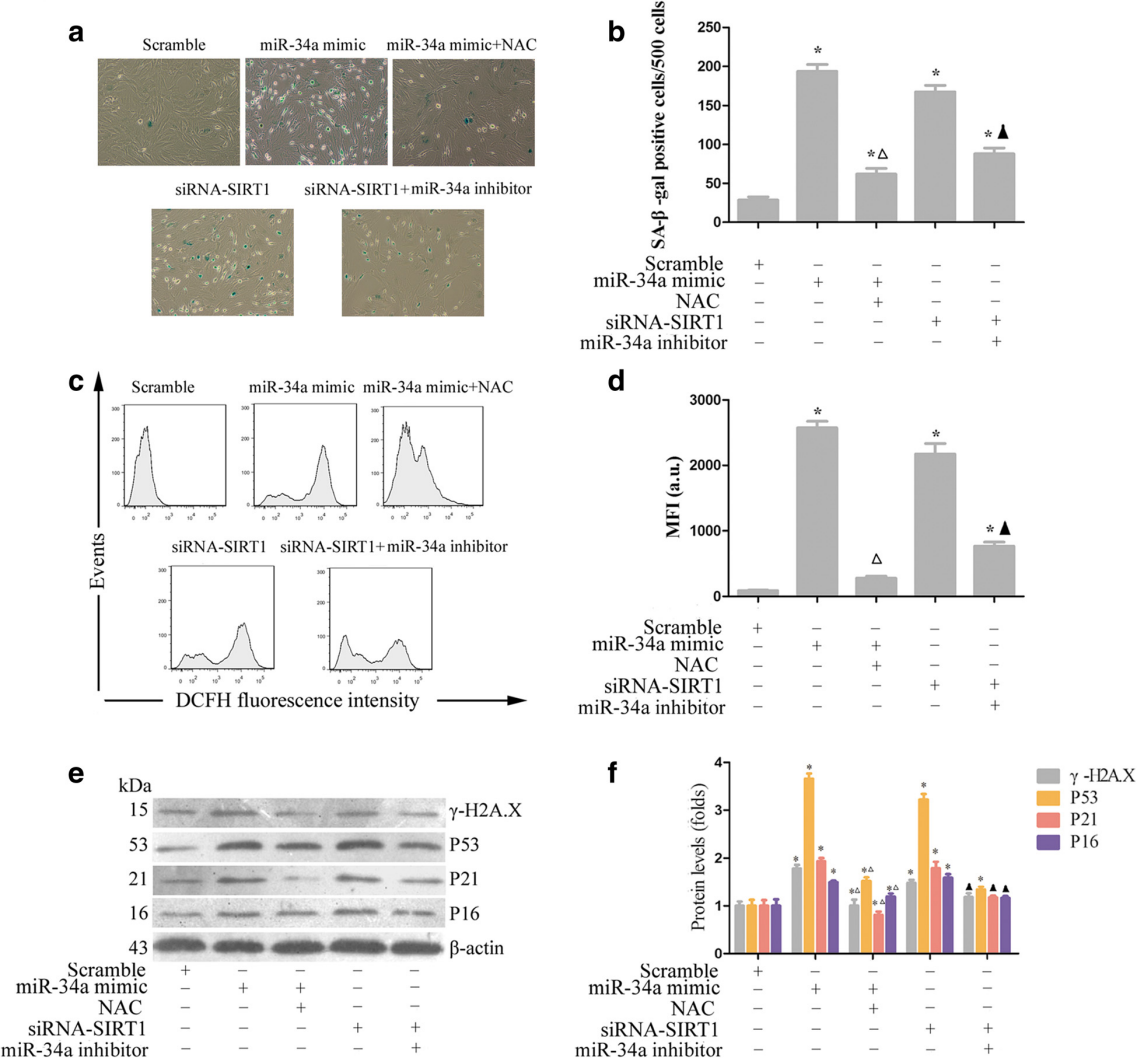
Overexpression of miR-34a induces senescence in MSCs. Cells were left untreated or pretreated with miR-34a mimic, siRNA-SIRT1, ROS scavenger NAC (10 mM), and miR-34a inhibitor separately or in combination for 72 hours, and then cellular senescence was analyzed by SA-β-gal staining (**a**, **b**). Cellular ROS production was assessed by measuring the fluorescent intensity of DCFH-DA determined using flow cytometry (**c**, **d**). Cellular DNA damage and senescence-related proteins including γ-H_2_A.X, p53, p21, and p16 were determined with western blot (**e**, **f**). Each column represents mean ± SD from three independent experiments. *P <0.05 vs. scramble, △*P* <0.05 vs. miR-34a mimic, ▲*P* <0.05 vs. siRNA-SIRT1.cp. *DCFH* 2′,7′-dichlorodihydrofluorescein, *MFI* mean fluorescence intensity, *miRNA* microRNA, *NAC N*-acetylcysteine, *SA-β-gal* senescence-associated galactosidase, *SIRT1* silent information regulator 1, *siRNA* small interfering RNA

Considering that the regenerative capacity of MSCs contributed greatly to their function, we further examined cellular senescence in miR-34a mimic-transfected MSCs. SA-β-gal activity, which is a characteristic feature of senescence-related growth arrest [31], was assayed. Results revealed that overexpression of miR-34a significantly increased the percentage of SA-β-gal-positive cells compared with that of scramble (Fig. 5a, b). SIRT1 inhibition has been reported to be associated with premature senescence and impaired proliferative activity in EPCs [17]. Consistently, the percentage of SA-β-gal-positive senescent cells was remarkably increased following SIRT1 knockdown (Fig. 5a, b).

ROS have been reported to induce oxidative stress that causes DNA and cell damage and can induce cell senescence through the p53/p21 pathway [32]. As expected, the miR-34a mimic increased ROS production, which was alleviated by addition of the ROS scavenger *N*-acetylcysteine (NAC) (Fig. 5c, d). γ-H2A.X, a sensitive marker for the formation of DNA damage foci, was examined by western blotting together with the senescence-related proteins p53, p21, and p16. In the miR-34a mimic and siRNA-SIRT1 group, the expression of p16, p53, and p21 was obviously increased compared with that in the control group (Fig. 5e, f). However, when ROS was removed by NAC or when miR-34a was inhibited by a miR-34a inhibitor, the expression of γ-H2A.X, p16, p53, and p21 was significantly reduced (Fig. 5e, f).

## Discussion

Our results show that miR-34a is significantly upregulated under H/SD conditions in MSCs, and overexpression of miR-34a is strongly associated with increased apoptosis, lower viability, and increased senescence. SIRT1, identified as a direct and functional target of miR-34a, protects MSCs from H/SD-induced apoptosis through its downstream effector FOXO3a. Further experiments indicated that the mitochondrial permeability transition and intrinsic apoptosis pathway of the CASP3–PARP1 axis were involved during this process. Moreover, miR-34a was also found to aggravate the senescence of MSCs in a ROS-dependent manner. This study strongly suggests that miR-34a is a promising candidate for a more optimized and appealing target for MSC-based therapy in MI.

Since MSCs are easily obtained and exhibit impressive paracrine ability as well as multilineage differentiation potential [33], autologous MSCs offer a great advantage when transplanted into ischemic or infarcted heart to regenerate and repopulate the injured myocardium and restore heart function. However, repair of cardiomyocytes and restoration of heart function are limited by poor survival [34], increased senescence [35], and loss of immunoprivilege in long-term preclinical studies of engrafted MSCs in the infarcted area [36]. Researchers have attempted numerous approaches to overcome these limitations, and have made some improvements in restoring cardiac function [37, 38]. Despite these successes, strategies are still needed to make transplanting MSCs into the infarcted area easier and more effective.

Recent observations have revealed that miRNAs are involved in the processes of apoptosis, proliferation, senescence, autophagy, and differentiation, among others, exhibiting powerful and unexpected roles in modulating cell biological functions by upregulating or downregulating these processes [18, 39]. Among the known miRNAs, miR-34a has been demonstrated to be involved in apoptosis [40] and senescence [17] and to inhibit various key regulators of cell cycle progression [41]. miR-34a belongs to one of several evolutionarily-conserved families of miRNAs, namely miR-34, and was originally identified as a TP53-targeted mRNA [40]. miR-34a was expressed in almost every tissue but was scarcely expressed in lung tissue [42]. Frequently downregulated and functioning as an independent prognostic indicator in multiple types of cancers [18, 43], miR-34a expression levels were significantly upregulated in the animal model of acute MI and in the aged hearts [15] and were strongly correlated with left ventricular end-diastolic dimension 1 year after acute MI [44]. Furthermore, overexpression of miR-34a was demonstrated to promote the apoptosis of myocardial cell during MI [45], aggravate senescence, and impede angiogenesis ability of EPCs [17] and endothelial cells [46], as well as induce senescence and inflammation in vascular smooth muscle cells [47], leading to myocardial and vascular dysfunctions. Delivery of antagomir Ant-34a or LNA-based anti-miRNAs, however, enhanced cardiac contractile recovery after acute MI, which was associated with reduced fibrosis, increased capillary density, and elevated myocardial cell functions [14, 15]. Thus, we presumed that miR-34a may play a crucial role in the MI microenvironment and contribute to the poor survival rate of MSCs in the infarcted area. In the present study, we showed that miR-34a was expressed in normal MSCs and was elevated greatly during H/SD, designed to mimic the in vivo conditions of ischemia and hypoxia. Overexpression of miR-34a aggravated MSC apoptosis, while inhibition of miR-34a expression conferred resistance to H/SD-induced apoptosis. These data suggests that miR-34a not only regulates resident myocardial cell apoptosis but also plays an important role in engrafted MSCs survival in the infarcted area.

SIRT1, one of the potential targets of miR-34a, has been identified as an apoptosis inhibitor and has been found to act as a longevity gene in many studies reported previously [48]. Recently, SIRT1 was reported to inhibit the apoptosis of vascular adventitial fibroblasts (VAFs) [49]. Consistent with this report, our results showed that when SIRT1 was knocked down by siRNA-SIRT1, the apoptosis of MSCs induced by H/SD increased. However, we found a moderate increase in the expression of SIRT1 protein level in MSCs during H/SD (Fig. 3e), which was also found in EPCs exposed to H_2_O_2_ [24]. As is well known, SIRT1 is an NAD-dependent deacetylase, and the balance between NAD^+^/NADH is crucial for cellular survival. A recent study demonstrated that during hypoxic and ischemic insults the concentration of NAD^+^ increases at the very beginning, leading to activation of the SIRT1-dependent cleavage of acetyl groups [50]. This compensation effect can partly explain our result of the increased SIRT1 expression during H/SD.

In response to oxidative stress, SIRT1 forms a complex with FOXO3 and then enhances cellular stress resistance [51]. FOXO3a is a member of the mammalian FOXO family of forkhead transcription factors, which are critical regulators of stress responses, oncogenesis, and longevity by directly regulating the genes involved in apoptosis, cell cycle progression, and stress responses [52]. FOXO activities can be regulated by phosphorylation, ubiquitination, as well as deacetylation to inhibit apoptosis [25, 51, 53]. It has been identified that SIRT1 can bind to and deacetylate FOXO3a and then suppress its transcriptional activity, which also exerts favorable effects on oxidative stress resistance in cardiac myocytes [51]. In the current study, we showed in MSCs that FOXO3a was downregulated in response to a miR-34a inhibitor, and could be abolished by silencing SIRT1 expression, suggesting that the restorative function of the miR-34a inhibitor in MSCs was mediated through the SIRT1–FOXO3a signaling pathway. Regulation of the SIRT1–FOXO3a signaling pathway through some basic strategies may therefore play an active role in clinical heart disease.

We further examined the role of ΔΨm and the intrinsic apoptosis pathway of the CASP3–PARP1 axis in the pro-apoptotic activity of miR-34a in MSCs. Considerable evidence implicated that mitochondrial dysfunction or a change in the ΔΨm was one of the signs of cell death [28]. The decrease of ΔΨm activates effector CASP3 by a series of reactions, and subsequently induces cell apoptosis. As expected, we found that inhibition of miR-34a increased the ΔΨm of MSCs during H/SD, and decreased cleaved CASP3 expression.

Like other cells, MSCs entered into the senescence process when exposed to oxidative stress which greatly reduced their regenerative capacity and limited their transplantation efficiency. As an inevitable by-product of mitochondrial respiration, ROS in moderate amounts is necessary for cell survival, proliferation, and longevity [54]. However, during hypoxia, an imbalance between the formation and scavenging of free radicals leads to overproduction of electrons. These electrons react with remnant molecular oxygen, leading to ROS generation [55]. Abundant ROS could result in cell senescence through inducing DNA damage [54]. In this study, we explored whether ROS was the main mediator of MSC senescence induced by miR-34a overexpression. Results showed that overexpression of miR-34a increased β-galactosidase-positive cells as well as ROS production. When ROS production was reduced by NAC in MSCs, DNA damage was attenuated and the expression of p16, p53, and p21 was reduced. These results imply that ROS has an important role in MSC senescence induced by miR-34a overexpression and thus may partly explain the poor survival rate of engrafted MSCs in the infarcted area.

With rapid development of anti-miRNA chemistries, even ahead of miRNA mimicry [56], the miR-34a knockdown by antagomirs or LNA-based anti-miRNAs has been shown to protect against the deterioration of cardiac systolic function in mice after acute MI [15]. Moreover, no side effect was reported. Intracoronary infusion or intramyocardial delivery of MSCs modified with current developing therapeutics to inhibit the expression of miR-34a might thus have great advantage in application for vascular diseases.

In conclusion, our study reveals that miR-34a is greatly elevated in MSCs during H/SD, and overexpression of miR-34a leads to robust apoptosis, while inhibition of miR-34a significantly increases pressure resistance of MSCs to H/SD. This apoptosis is highly regulated by SIRT1/FOXO3a pathway activation, mitochondrial dysfunction, and finally activation of the intrinsic apoptosis pathway of the CASP3–PARP1 axis. Moreover, our results show that miR-34a overexpression increases cellular senescence, which may be regulated by ROS production. However, more work will be needed to determine the role of SIRT1 and FOXO3a in miR-34a-mediated functions in myocardial ischemia in vivo and to investigate whether other signaling pathways such as NOTCH signaling pathways, which have been reported to be regulated by miR-34a [57], are involved in the H/SD process in MSCs.

## Conclusions

Our data demonstrate that miR-34a is involved in the process of H/SD in MSCs, while inhibition of miR-34a leads to an increase in SIRT1 and a decrease in FOXO3a protein expression, fewer apoptotic cells, and better viability. Moreover, we found that overexpression of miR-34a induced senescence of MSCs, which may partly be abolished by the ROS scavenger NAC. Inhibition of miR-34a in MSCs would thus be beneficial and could demonstrate great therapeutic potential in clinical transplantation for vascular disorders.

## Abbreviations

∆Ψm: Mitochondrial membrane potential
BCA: Bicinchoninic acid assay
CASP3: Caspase 3
CCK-8: Cell counting kit-8
DCFH-DA: 2′,7′-Dichlorodihydrofluorescein diacetate
EPC: Endothelial progenitor cell
FBS: Fetal bovine serum
FITC: Fluorescein isothiocyanate
FOXO3a: Forkhead box O transcription factor 3a
GAPDH: Glyceraldehyde 3-phosphate dehydrogenase
H/SD: Hypoxia and serum deprivation
IHD: Ischemic heart disease
IMDM: Iscove’s modified Dulbecco’s medium
MI: Myocardial infarction
miRNA: MicroRNA
MSC: Mesenchymal stem cell
NAC: *N*-acetylcysteine
NC: Negative control
PARP1: polyADP-ribose polymerase 1
PBS: Phosphate-buffered saline
PI: Propidium iodide
PVDF: Polyvinylidene difluoride
qRT-PCR: Quantitative RT-PCR
ROS: Reactive oxygen species
SA-β-gal: Senescence-associated β-galactosidase
SD: Standard deviation
SDS: Sodium dodecyl sulfate
siRNA: Small interfering RNA
siRNA-NT: Scrambled siRNA
SIRT1: Silent information regulator 1
TBS: Tris-buffered saline
TBS-T: TBS with Tween-20
UTR: Untranslated region
VAF: Vascular adventitial fibroblast
