# Correction to: Roles of microRNA-34a targeting SIRT1 in mesenchymal stem cells

**DOI:** 10.1186/s13287-020-01854-w

**Published:** 2020-07-31

**Authors:** Fengyun Zhang, Jinjin Cui, Xiaojing Liu, Bo Lv, Xinxin Liu, Zulong Xie, Bo Yu

**Affiliations:** 1 Key Laboratories of Education Ministry for Myocardial Ischemia Mechanism, The Second Affiliated Hospital of Harbin Medical University, 148 BaojianRoad, Harbin, 150086 P.R. China; 2 Department of Cardiology, The SecondAffiliated Hospital of Harbin Medical University, 148 Baojian Road, Harbin, 150086 P.R. China; 3 Department of Cardiology, Mudanjiang Forestry CentralHospital, 50 XinhuaRoad, Mudanjiang, 157000 P.R. China

**Correction to: Stem Cell Res Ther 6, 195 (2015)**

**https://doi.org/10.1186/s13287-015-0187-x**

The original article [1] contains an error whereby the authors found that they incorrectly duplicated the primer sequence of Sirt1 over the primer sequences of miR-34a and U6.

Zhang et al. apologise for this mistake, and note the correct version of Table 1 ahead in this Correction article with corrected primer sequences for miR-34a and U6.

**Table 1:** Primers for qRT-PCR and oligonucleotide

Name		Sequence
**Quantitative RT-PCR**		
miR-34a		5′-UGGCAGUGUCUUAGCUGGUUGUU-3’
SIRT1	Forward	5′-AAGGCCACGGATAGGTCCATA-3’
	Reverse	5′-CGCTTTGGTGGTTCTGAAAGG-3’
FOXO3a	Forward	5′-TGCCGATGGGTTGGATTT-3′
	Reverse	5′-CCAGTGAAGTTCCCCACGTT-3′
U6	Forward	5′-CCTGCTTCGGCAGCACA-3’
	Reverse	5′-AACGCTTCACGAATTTGCGT-3’
β-actin	Forward	5′- CCCAGCACAATGAAGATCAAGATCAT −3’
	Reverse	5′- ATCTGCTGGAAGGTGTACAGCGA − 3’
**Oligonucleotide**		
miR-34a mimic		UGGCAGUGUCUUAGCUGGUUGUU CAACCAGCUAAGACACUGCCAUU
NC mimic		UUCUCCGAACGUGUCACGUTT ACGUGACACGUUCGGAGAATT
miR-34a inhibitor		UGGCAGUGUCUUAGCUGGUUGUU
NC inhibitor		CAGUACUUUUGUGUAGUACAA
siRNA-SIRT1		GCACCGAUCCUCGAACAAUTT AUUGUUCGAGGAUCGGUGCTT
siRNA-NT		UUCUCCGAACGUGUCACGUTT ACGUGACACGUUCGGAGAATT
